# *“It Is Good for My Family’s Health and Cooks Food in a Way That My Heart Loves”*: Qualitative Findings and Implications for Scaling Up an Improved Cookstove Project in Rural Kenya

**DOI:** 10.3390/ijerph9051566

**Published:** 2012-04-30

**Authors:** Bobbie Person, Jennifer D. Loo, Mercy Owuor, Lorraine Ogange, Maria Elena D. Jefferds, Adam L. Cohen

**Affiliations:** 1 Office of the Director, National Center for Zoonotic and Emerging Infectious Diseases, Centers for Disease Control and Prevention,1600 Clifton Road, Atlanta, GA 30333, USA; 2 Respiratory Diseases Branch, National Center for Immunization and Respiratory Diseases, Centers for Disease Control and Prevention,1600 Clifton Road, Atlanta, GA 30333, USA; Email: ihi4@cdc.gov (J.D.L.); cohena@sa.cdc.gov (A.L.C.); 3 Kenya Medical Research Institute, Box 1578, Kisumu, Kenya; Email: owuormercy@gmail.com (M.O.); archielaogange@yahoo.com (L.O.); 4 International Micronutrient Malnutrition Prevention and Control Program, Divisions of Nutrition, Physical Activity and Obesity, Centers for Disease Control and Prevention,1600 Clifton Road, Atlanta, GA 30333, USA; Email: mnj5@cdc.gov

**Keywords:** improved cookstoves, developing countries, indoor air pollution, formative research

## Abstract

The use of indoor, three-stone fire pits in resource–poor countries is a substantial burden on human health and the environment. We conducted a pilot intervention promoting the purchase and use of an improved cookstove in rural Kenya. The goals of this qualitative inquiry were to understand the motivation to purchase and use; perceived benefits and challenges of cookstove use; and the most influential promotion activities for scaling up future cookstove promotion. Purposive sampling was used to recruit 10 cookstove promoters and 30 cookstove purchasers in the Luo community. Qualitative semi-structured interviews were transcribed and a thematic analysis conducted. Women reported the need for less firewood, fuel cost savings, reduced smoke, improved cooking efficiency, reduced eye irritation, lung congestion and coughing as major benefits of the cookstove. Cost appeared to be a barrier to wider adoption. The most persuasive promotion strategies were interpersonal communication through social networks and cooking demonstrations. Despite this cost barrier, many women still considered the improved cookstove to be a great asset within their household. This inquiry provided important guidance for future cookstove implementation projects.

## 1. Introduction

Globally, indoor smoke from biomass fuels ranks eighth as a risk factor for burden of disease and in developing countries is associated with 4–5% of all deaths and disability-adjusted life-years lost [1]. An estimated 70% of the world’s poorest 1.3 billion people are women who rely on biomass energy, in the form of firewood, crop residues, and charcoal, burned in traditional and inefficient cookstoves [2]. More than an estimated 1.5 million deaths in children <5 years of age worldwide are caused by acute respiratory infections, and exposure to indoor smoke, often originating from inefficient cookstoves used for cooking and boiling water for drinking, more than doubles the risk of pneumonia [3,4,5].

Exposure to indoor air pollution is an important public health problem in rural Western Kenya as most households cook with open pit three-stone cookstoves that use biomass fuels and women and children in these households experience a daily median of five hours of exposure to indoor air pollution from cooking [6]. A survey conducted in Nyanza Province, western Kenya, in March-April 2007, found a heavy burden of respiratory illnesses, with caregivers reporting that 22% of children aged 6–35 months experienced an acute respiratory infection (defined as cough or difficulty breathing in the last 24 hours) in the past two weeks [7]. Effective strategies to improve indoor air quality include use of cleaner-burning, efficient cookstoves. Reducing exposure to indoor air pollution through cleaner-burning cookstoves represents one WHO-recommended intervention to reduce respiratory diseases and improve the health of women and children who spend more time around the hearth of the home [8,9].

In an effort to mitigate the negative effects of traditional three-stone and inefficient charcoal cookstoves, we initiated a pilot cookstove improvement project among poor Luo communities in Nyanza Province in Western Kenya**. **The project was conducted in 10 rural villages from July 2008–March 2009, in conjunction with the Safe Water and AIDS Program (SWAP), a Kenyan non-governmental organization that trains HIV self-help and support groups to provide health education and sell health products to their community members as an income generating activity in rural villages and peri-urban slums [7]. SWAP group members receive training on evidence-based health products (such as safe water systems, insecticide treated bednets, and soap), health education and promotion strategies, and microfinance skills. Following trainings, individual members take on the role of SWAP health promoters carrying out peer-to-peer health promotion and selling health products in their communities for a profit. 

**Figure 1:**
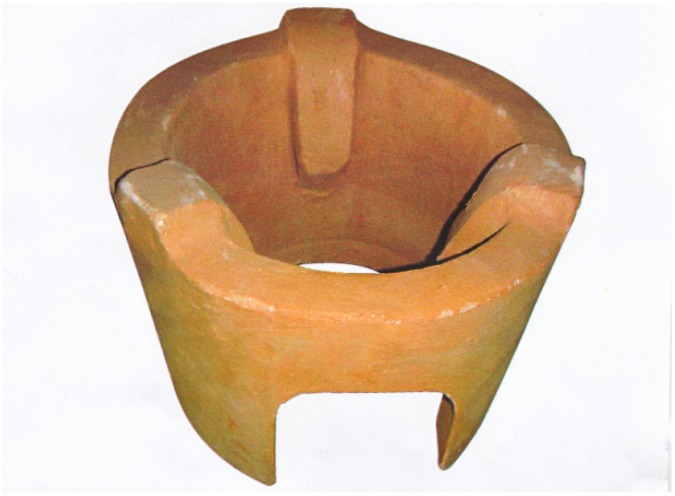
Upesi jiko, locally manufactured ceramic liner.

For the improved cookstove project, SWAP health promoters in the 10 villages received additional training on the prevention of respiratory illnesses and promotion of an improved cookstove, the upesi jiko, as well as how to permanently install it in the purchasers’ home. The upesi jiko (Swahili for “quick cookstove”) is a simple ceramic liner installed into a simple, earthen base that is constructed semi-permanently within a kitchen (Figure 1). The upesi jiko is locally produced by skilled pottery groups guided by the Kenya Bureau of Standards (KS 1814:2005) to ensure that the dimensions of the ceramic liner are energy efficient [10]. The cookstove costs 150 Kenyan Shillings (Ksh), or ~$2.00 U.S. Dollars (USD). Additional material and labor costs for the installation of the liner into a permanent earthen base typically added $1.50 to $3.00 USD to the purchase. Some testing has been conducted in both lab and field settings; however, findings from these evaluations have not yet been published.

Our study had three major components. We assessed current cookstove practices and approaches to adoption of the locally manufactured cookstoves. Additionally, we assessed the efficacy of the stove in reducing harmful emissions. Findings from these studies will be presented elsewhere. We also explored the actual experiences of stove promoters in persuading women to purchase and use the stove, as well as the actual experiences of women who purchased a stove and assessed what influenced their purchase and use of the improved stove. We present those narrative findings in this report. We explored from the SWAP health promoter’s perspective: the most effective cookstove promotion activities, community reactions to the cookstove intervention, perceptions of the SWAP cookstove trainings, and benefits experienced by using the new improved cookstove. In addition, we wanted to better understand from the purchaser’s perspective: their motivation to purchase and install the improved cookstove, how they secured the funds to purchase the improved cookstove, the benefits and challenges of the cookstove use, and other factors that influenced their experiences with or ability to use the upesi jiko. 

## 2. Methods

### 2.1. Study Area and Participants

Our study was conducted in Nyanza Province in rural Western Kenya between July 2008 and March 2009, across rainy and dry seasons. Nyanza is one of Kenya’s poorest areas, with an estimated 63% of the population living on less than $1 U.S. a day [11]. It also has among the lowest life expectancies in the country—age 43 for women and 37 for men—and one of the worst child survival outcomes in Kenya, with 133 infant deaths per 1,000 live births and an under-five mortality of 206 per 1,000 [11]. Only 6% of the population has access to piped water and 5% access to electricity [11]. The majority of the population are Luo subsistence farmers who cultivate maize, sorghum, cassava, and millet; carry out animal husbandry and subsistence fishing; and engage in migrant labor. Families in this polygamous society often live in multigenerational compounds (“dalas”) that consist of a single main house surrounded by one to three additional households [12]. Households in this area include a mixture of structures. Some housing structures consist of one large area where both cooking and sleeping occur while other housing structures have multiple rooms in which the cooking area is separate from the living and sleeping areas. Additionally, there is a small subset of houses that have a completely separate housing structure for cooking.

### 2.2. Design and Data Collection

Purposive sampling, a method of sampling in which participants are selected because of a certain characteristic, was used to recruit 10 SWAP health promoters who owned and were selling the new upesi jiko along with 30 Luo women who had purchased and were using the upesi jiko for in-depth, key informant interviews [13,14,15]. Semi-structured, in-depth interviews were conducted in Dholou by two Luo bilingual qualitative research assistants from July 2008–March 2009. Data collection was facilitated by interview guides with pretested open-ended questions and probes. Bilingual project staff previously trained in qualitative data collection methods by a CDC senior qualitative researcher, translated questions and probes into Dholou. Questions were then pretested with local women to assure correspondence with the local dialect, cultural meanings, and women’s understanding. Interview guides focused on experiences with costs, decisions to purchase an improved cookstove, and benefits as well as challenges of using the cookstove. Verbatim notes were handwritten during interviews. Interviews were conducted over six months, allowing for constant comparison so that our preliminary data analysis could help guide subsequent interviews. The 40 interviews, approximately 1 hour in length, took place predominately in women’s homes in rural Kenya. Institutional and ethical review boards from the Centers for Disease Control and Prevention (CDC) and Kenya Medical Research Institute, Kisumu, Kenya, approved this study.

### 2.3. Data Management and Analysis

Interviews were hand-written and transcribed verbatim, translated into English by bilingual research assistants, and then entered as a Microsoft Word^©^ document into ATLAS-ti^©^ to facilitate text searching, data coding, and analysis [16]. We used a modified grounded theory approach for data analysis [13,17]. This approach is designed to discover theory within narrative data. The primary author used open, axial, and selective coding to analyze the narratives. Open coding, a word-by-word analysis, was used to identify, name, and categorize events and explanations of the day-to-day reality of participants related to cookstove use [13,18,19]. Categories were related to other categories and guided further exploration into variances and similarities within and across the narratives [19]. 

## 3. Results

### 3.1. Characteristics of Participants

Women had been using the stove a range of two weeks to eight months at the time of interview. The demographic characteristics of SWAP health promoters, who all came from the community, were similar to cookstove purchasers. All 40 participants were female; 93% (37/40) were married, 7% (3/40) widowed or separated, and 40% had one or more co-wives. Few women reported any secondary school education. The average number of children per participant was five children (range of 0–11). 

### 3.2. Use and Perceived Problems of Traditional Cookstoves

The traditional cookstove used in most homes within the Luo community is an open fire pit with three large stones placed around it to hold cooking pots. These fire pits are often used inside and without any mechanical ventilation. SWAP health promoters and women who purchased cookstoves reported that the traditional cookstove used large quantities of firewood and that securing sufficient firewood was often expensive and becoming increasingly difficult. This resulted in women and children spending a great deal of time and labor collecting firewood:

*Here in Nyando there is a problem with finding firewood and there are times when I do not have money to buy firewood. My child who is 14 years old has the responsibility of collecting firewood. He gets help sometimes from the younger siblings. The old cookstove used a lot of firewood. It is hard work. (cookstove purchaser)*

Women also reported that the traditional cookstove emitted a great deal of smoke that irritated their eyes, nose, and lungs leading to illnesses that often required money for healthcare:

*With the old cookstove you would have difficulty in breathing and cough a lot as a result of too much smoke. One gets itchy eyes that turn red. You have difficulty in breathing if there is a lot of smoke in the kitchen. You will also have a runny rose which can also lead to headaches. I know that when you have difficulty in breathing then it means that your lungs have failed and they are not functioning well. (cookstove purchaser)*

Additionally, women reported that the traditional cookstoves burned too hot and erratically and created soot and ash that often landed in their food and dirtied their home. Women and children also suffered burns as a result of the open fire flaring up, when stoking the fire, or when removing a hot cooking pot. There was also a fear that their home could burn to the ground if the cookstove was not watched.

Most women cooked inside of the home. About a quarter of women cooked in an area where household members slept. Cookstove use was influenced by seasonality, weather, availability of cooking fuel, and number of people being cooked for. During summer months, some women constructed a three-stone cookstove outside of the house for cooking to reduce heat in the home and then constructed the cookstove back inside when cooler weather approached. A few women supplemented cooking for large crowds with simple portable paraffin or kerosene cookstoves but the increased expense often prevented regular use of these cookstoves in the home. Approximately one-third of women reported using their three-stone stoves in addition to their improved stove when cooking for large gatherings. 

### 3.3. Considerations When Purchasing a New Cookstove

Lengthy consideration was often given when purchasing an expensive household item, such as a cookstove. Women who purchased the upesi jiko often identified themselves as very poor with little discretionary income, and burdened by uncontrollable life circumstances:

*We are always looking out for money to feed our families. I had planned on taking a tailoring course but I haven’t because my hands are short (lack money). We are a poor community. (cookstove purchaser)*

Drought and famine were mentioned as contributing to their economic hardships and that of their neighbors:

*So many people are longing to have the cookstove but the price is so expensive. People cannot afford it now with the drought. The hunger has hit us hard. You know we are faced with very hard economic times. People don’t have maize in their stores to scoop anytime they want to. There is famine and people are crying about the price. (cookstove purchaser)*

The ability of women to purchase an upesi jiko varied across women and was related to their access to cash and position of power in the household related to their status (for example as first wife, favored wife, or mother-in-law) within these polygamous multigenerational households. Some women chose to use their own money to purchase the upesi jiko (money often being saved to purchase other items), or acquired it through a community lending scheme with other villagers. Some women reported that monies saved to purchase a new dress, new shoes, new pot, maize flower, food for the day, and other commodities were diverted to purchase a cookstove:

*You know women are very clever. They can save a little from the food money each time and find themselves having a lot more than their husbands expect. They end up having money when times are hard up. I had saved up to buy shoes and decided to buy the cookstove instead. (cookstove purchaser)*

Many women described securing the funds to purchase the cookstove as a negotiation with their husband, co-wife or mother-in-law, with some negotiations faring better than others:

*My mother-in-law was the one who negotiated it for me. She told my husband that the cookstove was really good and she would like us to install one in her kitchen and mine so that we would have an easy time for cooking. (cookstove purchaser)*

While most husbands were viewed as receptive to the woman purchasing an upesi jiko, several women said that they had no power to make such a decision. Resources in polygamous households, divided among 2 or 3 wives and numerous children, were often controlled by either the “first wife” or the ‘favored wife.” Most of the women reported having a favorable relationship with their co-wife and often encouraged the husband to buy the other wife household items or shared theirs. Second younger wives often deferred decisions to the older first wife.

### 3.4. Reported Affordability of the Improved Cookstove

Women reported that the main advantage of the traditional cookstove was that it was free. Overall, women reported they did not think the cost of purchase and installation of an upesi jiko was affordable to most families in their communities:

*People in the village, we are saying that the new cookstove is really good and they would all love to install one. But we are crying that we do not have money. There is inflation that has hit hard and the money is very little. (cookstove purchaser)*

The new cookstove, including installation by the SWAP health promoter, generally costs between 200–300 ksh ($2.50–3.50 U.S.) with an additional burden of 50–100 ksh to acquire the necessary materials for installation. Women felt that people who engaged in paid labor were more likely to be able to purchase a cookstove, but that very few people were paid for work. Several women also talked about a similar, cheaper cookstove in the market that could be purchased for 50 ksh but they had heard that it would often crack. Many people requested SWAP promoters to reduce the price or provide them a cookstove on credit.

### 3.5. Reported Household Benefits of Improved Cookstove Use Among Women Who Purchased Cookstoves

Almost all women described the major benefits of their new cookstove as the need for less firewood resulting in cost savings as well as reduced smoke in their homes:

*We buy firewood. With the old cookstove I could use fuel worth 70/ksh- a week and now I only spend 30/ksh worth of firewood a week. (cookstove purchaser)*

In addition, they described the benefits to their cooking experience and to the kitchen environment. Women explained that their kitchens were now “tidy” because soot and ash no longer flew up into their food and the overall appearance of the kitchen area was more appealing. Women also described that their food cooked faster and more evenly, pans were steadier, the sides of the cookstove served as counter space for their pots and pans, and they sustained fewer burns on their hands. Furthermore, women reported that the cookstove was more efficient, retained heat longer, and made their work easier:

*The new cookstove makes my work easier. When I light the fire and put enough firewood, I don’t have to be there to keep on adding firewood to the fire. When you put food onto it, the food will cook, even when you have gone out to collect water from the river the food will not burn. (cookstove purchaser)*

Women also talked about unexpected advantages, such as how the cookstove stays dry during the rainy season, reduced back pain from not having to bend over the cookstove during cooking to blow on the fire, and the elevated status of their household in the community due to their purchase of a cookstove.

### 3.6. Reported Health Benefits of Improved Cookstove Use Among Women Who Purchased Cookstoves

Women talked at length about the health benefits of reduced smoke for themselves and their children. They reported that the reduction of smoke in the house decreased irritation of their and their children’s eyes, runny noses, coughing, chest discomfort, and difficulties in breathing along with cost savings due to fewer hospital visits:

*It also has less smoke and the homa (runny nose, red eyes and breathing difficulties) that I used to have when I was cooking with the old cookstove is gone. The smoke leaves through the open air space on the wall above the cookstove. With the old cookstove you would have difficulty breathing and cough a lot as a result of too much smoke. The new cookstove is good and it protects the health of the family. I no longer spend a lot of money on taking my child to the hospital to be treated for coughs, running nose and red eyes with the new cookstove. (cookstove purchaser)*

Additionally, many women reported that the upesi jiko reduced burns and the workload of children. Overall, immediate household and health benefits were reported more frequently and appeared to have greater value than the consideration of long term health benefits.

### 3.7. Barriers to Proper Cookstove Installation

Several women bought the cookstove but delayed installation, explaining that the additional cost for installation, cost, and supplies were major barriers:

*Getting materials for installation is not easy. Murram [a type of rock], pebbles, and stones are not easy to get because they are expensive. When we purchase the cookstove there is no money left for these items. (cookstove purchaser)*

Permanent installation in the kitchen was a separate cost and women with fewer resources sometimes delayed installing their cookstove until they could save up the amount needed for installation supplies. Some women purchased a cookstove to install in a kitchen that was yet to be built. A couple of women explained that their husbands did not want them to create a vent, by cutting a hole in their wall, so while they were using the cookstove it wasn’t installed properly. After installing an upesi jiko, some women were reticent to discard their traditional cookstove because they wanted the ability to use it when necessary to cook for larger groups of family and friends.

### 3.8. SWAP Health Promoters

SWAP health promoters described numerous benefits of being part of SWAP and selling improved cookstoves to their community members. The benefits included the ability to bring needed health information and products to their community; opportunities to train others on health issues; opportunities to know and make a difference in their community; a chance for social connectedness; an increased sense of status in their community; and opportunities to increase their income:

*As SWAP vendors we are able to sell the SWAP products and earn money. We sit down as a group and decide on what to do with the profit. Sometimes we use the money to buy more products or simply to share the profit. When things are good and the sales are high, we are able to spare some money to buy some more products while at the same time leaving some to be shared as profits. (From) this I could even buy ¼ kg of sugar for my family. I no longer have to beg my husband for money! (SWAP health promoter)*

The SWAP health promoters reported that they used multiple techniques to promote the purchase and use of the upesi jiko in their communities. The SWAP health promoters most frequently told people that the cookstove used less fuel, cooked faster, produced less ash, saved money, and reduced smoke.

*I tell a person that the very first benefit is that it uses less firewood as compared to the 3 stones jiko cookstove. Secondly you can use any size of cooking pan on it because even a small or big size cooking pan can be used [on] it. Thirdly it has less smoke and as a result one doesn’t suffer from diseases of the chest when s/he is using this type of cookstove. (SWAP health promoter)*

Cookstove promotion activities typically occurred during community-based meetings, in the homes of SWAP promoters, walking door-to-door in villages, or in market settings. The SWAP health promoters explained that the most persuasive selling strategy was to have women visit them in their home and see them cooking on their cookstove:

*Many people come to my home to see the upesi jiko and because of the good things they see about it, this makes them like it. These are the people who purchased it because I was among the first ones to install it. Observing it is what makes people to like it and want to buy it. (SWAP health promoter)*

They reported that transporting the cookstoves and helping people find the necessary supplies to install the cookstove were the main challenges promoting the cookstoves along with turning down people wanting a cookstove on credit.

### 3.9. Community Interactions with SWAP

Women who purchased cookstoves reported they were motivated by the SWAP health promoter discussions describing their own cooking experiences, the potential cost savings, and health benefits of the improved cookstove. Many who purchased cookstoves spoke highly of educational sessions provided by SWAP health promoters, especially when accompanied by a cooking demonstration on the cookstove:

*There was cookstove training for the community that I attended. I was very excited and attended the demonstration done in one of the houses. I learned a lot. The people (SWAP vendors) are very smart and share what they know with you. I learned about the cookstove and how to install it. They taught us everything, the whole mixing process and how to make the slab. I bought one that day and had it installed the very day after the training. (cookstove purchaser)*

Purchasers consistently reported experiencing similar benefits of the cookstove as told to them by different vendors, most notably savings on fuel, retains heat longer, cooks food faster, produces little smoke, and reduces respiratory problems such as homa (a Luo word for runny nose and eyes). Women commented on the value of the new information and products the SWAP health promoters provided to the community. According to purchasers, economic savings associated with the new cookstove was a persuasive argument used by SWAP health promoters but seeing someone cooking on the cookstove convinced many to actually purchase the cookstove:

*I saw the cookstove at her house, this friend of mine. She was cooking and said that this thing is very good and every woman with a proper straight mind should have one. You do not disturb yourself with pushing in so much firewood. It keeps on burning. Seeing the cooking, it opened my mind! (cookstove purchaser)*

## 4. Discussion

There is a growing recognition for the need to understand which public health interventions work and how to make them succeed in the real world. Qualitative research utilizes a range of data collection approaches in a real life setting, which can inform health intervention projects in resource-poor countries, guide decision-making, assist in program design, explain behaviors, and better understand why programs succeed or fail [20,21,22]**. **This is especially important for public health interventions that reduce indoor air pollution, since the Global Alliance for Clean Cookstoves led by the United Nations was formed to promote cleaner burning cookstoves [23].

Findings from our qualitative inquiry provided valuable insights into both the SWAP health promoter and the cookstove purchaser and important information that was used in scaling up the intervention to a wider area. We determined that it was important to subsidize the upesi jiko and combine the purchase and installation cost. In addition, our findings allowed us to focus our messages on identifying community motivators along with emphasizing the role of cooking demonstrations to promote adoption of the upesi jiko. Similar to other studies, all participants identified problems with the traditional cookstove and reported considerable benefits of the improved cookstove to both their household environment and health [24,25,26]. Economic constraints profoundly affected participants’ day-to-day reality and income generated from promoting and selling cookstoves as well as income saved from using the cookstoves was seen as the greatest relative advantage over the traditional cookstove and a critical motivator for both promoters and purchasers. While health benefits were addressed, promoters more often focused on economic benefits of the improved cookstove due to the saliency of the topic to community members. Based upon the perceived local income levels of the community, the cost of the improved cookstove (150 Kenyan Schillings (Ksh) for purchase and 150 Ksh for installation—estimated $3 U.S.) was determined collectively by SWAP health promoters prior to the pilot. During implementation of the pilot, however, the SWAP health promoters determined that the cost was too high for many community members as the separate costs for purchase and installation of the improved cookstove often posed a barrier; use was often delayed for lack of money or supplies to finish installation. Cost is often a large factor in deciding whether to implement an intervention, especially in lower income communities. The literature regarding cost decisions for cookstoves is relatively limited and somewhat incomparable to literature regarding costs for other household health interventions as cookstoves tend to be more expensive and therefore, not as readily accessible for immediate purchase.

The intersection of drought, famine, and recent political instability, along with the household demands of potentially multiple wives and numerous children within a household compound often strained economic resources and limited women’s ability to purchase improved cookstoves. This is consistent with the findings of others who note that often the solutions to energy poverty are beyond the means of many in disproportionately poor populations [27].

As often seen in patriarchal societies in resource-poor countries, in this study, socially constructed roles for women related to household management, child care, and subsistence farming along with the additional requirement of searching, often for hours, for scarce biomass fuel increased the harsh burdens of day-to-day life among women [28]. Decision-making for household purchases, while often at the discretion of a woman, was nevertheless shaped by gendered power relationships with the husband and any co-wives, which is consistent with a similar study on improved cookstove use [26]. Men did not cook or collect fuel, and thus were less directly affected by the use of traditional cookstoves compared to women, which likely resulted in a lower motivation to purchase improved cookstoves for their wife (ves). For women, purchasing a cookstove with their own money often resulted in not purchasing other valuable household commodities such as food and clothing. These findings show that Luo women perceive the improved cookstove as a valuable product, but also highlight how their decision to purchase an improved cookstove can further exacerbate their economic vulnerability. This is an important factor to consider in determining pricing and promotion strategies for wider implementation of improved cookstove promotions.

Similar to findings from other qualitative studies on improved cookstove use, women in both groups reported that the use of inefficient traditional cookstoves resulted in numerous negative health consequences (irritated eyes, coughs, runny noses, difficulty breathing, and other respiratory illnesses) for themselves and their children, which then decreased when using an improved cookstove [29,30,31]. Furthermore, the need for less wood due to the improved efficacy of the cookstove reduced not only the labor and time required to search for and carry such wood but the accompanying joint pain, muscle strain, and other negative health effects which disproportionately affect women and children.

Consistent with the behavioral constructs of role modeling, observational learning, and self-efficacy, the most persuasive cookstove promotion strategy was that of seeing someone cooking on the jiko and discussing its economic and health benefits for women and the household [32]. Women who purchased the cookstove often talked with other women about their cookstove and described its numerous positive benefits, highlighting the critical role of interpersonal communication through social networks as primary to persuasive communication in the community setting. Health promoters found value in being part of SWAP along with the corresponding health education and health products they were able to offer the community.

As is shown in this study, qualitative inquiry is mainly an inductive rather than deductive analytical process concerned with the subjective experiences of participants around specific phenomena under study, such as the upesi jiko. Findings derived from qualitative formative data collection allowed for the scaling up of the pilot cookstove intervention in a locally relevant and appropriate way. While our findings showed that the upesi jiko was generally well received for various reasons, many of our participants noted that cost was an important barrier in the adoption of these cookstoves. Knowledge of this potential barrier to implementation allowed us to subsidize the cookstove as plans moved forward to scale up the project. The most salient pervasive factors for purchasing a cookstove were the economic savings and the perceived reduction of more immediate health effects of smoke to women and their children when cooking. Continuing to promote community identified motivators along with the potential long term health benefits will be critical in a community where long term health benefits may not be as salient as day-to-day concerns.

Previous studies have noted the importance of culturally relevant promotion and marketing strategies to effectively implement an improved cookstove intervention [24,33,34,35]. In our study, women described interpersonal communication through social networks of people they trusted, along with actual demonstrations of the cookstove in use as major influences on their decision to purchase and use the cookstove. The use of SWAP health promoters, who are members of the community and actively involved with local groups and individuals, will continue as a primary cookstove promotion approach with an emphasis on actual cookstove use demonstration. Enhancing the skills of SWAP health promoters and expanding the social network approach will allow for the promotion of the cookstoves throughout the community in a strategic, community-tailored approach. In addition, enhancing promotion activities to include male decision-makers may influence household discussion of purchasing an improved cookstove.

Our study has some limitations. Although, we believe that our findings would be transferable to poor populations near our study community with similar cultural characteristics and resource limitations, it may be less relevant to urban or nomadic populations in other parts of Kenya. We limited our inquiry to women who had purchased a cookstove. Additional inquires among women who had not purchased the cookstove and with men who are often in charge of household spending would have provided a more robust inquiry especially in resource-poor communities that have multiple demands on household income. Furthermore, all of the women in our study had recently purchased a cookstove and the women’s responses might have changed after a longer period of stove ownership. Lastly, some of our findings were based on self-report and not direct observation, which might have biased our findings if women responded with socially desirable answers.

## 5. Conclusions

Qualitative research allowed us to better understand the social setting, economic constraints, and complex social and gender processes surrounding household decision-making, as well as the perceived benefits and challenges of the improved cookstove in a rural Kenya community. These findings will be used to appropriately tailor future cookstove promotion activities in this disproportionately poor Luo community. Findings from our qualitative inquiry provided important guidance to program design and community promotion activities for scaling up a successful pilot cookstove improvement project. Qualitative research should play an important role in the scaling up of successful health promotion projects to ensure cultural relevance and health equity.
